# Clinical and biological correlations in acute toxoplasmosis

**DOI:** 10.1186/1471-2334-14-S7-P15

**Published:** 2014-10-15

**Authors:** Andrei Csep

**Affiliations:** 1 Faculty of Medicine and Pharmacy, University of Oradea, Romania

## Background

Toxoplasmosis is a zoonosis caused by a coccidian protozoan, *Toxoplasma gondii*.

## Methods

The study included 407 non-pregnant women with high risk for toxoplasmosis, presented to the Infectious Diseases Clinic in Oradea in the period 01.01.2009-31.12.2012. By MEIA (Microparticle Enzyme Immunoassay) performed in Bioclinica laboratories, values of Toxoplasma IgM and IgG antibodies were determined and by EIA (Enzyme Immunoassay), the values of IgA Toxoplasma antibodies, as well as the dynamic tracing of their evolution over a period of 12 month.

## Results

The results of investigations showed that 24.3% of non-pregnant women had acute toxoplasmosis, 25.6% had acute toxoplasmosis in their past, at a rate of 60% the serology was completely negative. Most cases of acute toxoplasmosis were diagnosed in spring and autumn (p=0.0373). The most affected range was the age group 21-25 and 26-30 (p<0.0001). The gynecologist and the patients’ own initiative has an important role in guiding them to make analysis (p<0.0001). The main reason for presentation to the Infectious Clinic was the appearance of adenopathies with cervical localization (p=0.0001). The optimum period of time necessary to achieve negative level of IgM and IgA *Toxoplasma* was between 3 to 6 months after presentation (p<0.0001).

## Conclusion

The infection with *Toxoplasma gondii* affects especially young women; most of the cases appear in spring and autumn.

